# Video Narrative Exposure Therapy (NET) with Children and Young People who Witnessed Domestic Violence: A Naturalistic Single Case Study Series

**DOI:** 10.1007/s40653-024-00681-y

**Published:** 2025-01-10

**Authors:** Fiammetta Rocca, Thomas Schröder, Nima Golijani-Moghaddam, Sarah Wilde

**Affiliations:** 1 Mental Health and Clinical Neurosciences Unit, School of Medicine, University of Nottingham, Nottingham, UK; 2 Nottinghamshire Healthcare NHS Foundation Trust, Nottingham, UK; 3 School of Psychology Sport Science & Wellbeing, University of Lincoln, Lincoln, UK; 4 Lincolnshire Partnership NHS Foundation Trust, Lincoln, UK

**Keywords:** Posttraumatic stress, Complex trauma, Narrative exposure therapy, KIDNET, Children and young people, Domestic violence, Remote psychotherapy

## Abstract

This study investigated the potential effectiveness, feasibility, acceptability, and putative mechanisms of change of Narrative Exposure Therapy (NET) delivered via videoconferencing with young people who witnessed domestic violence. A naturalistic, mixed-method, AB, interventional single case design was used. Five female adolescents aged 13–17 years were recruited from a Child and Adolescent Mental Health Service in the United Kingdom and attended 4–10 video-sessions of the child-friendly NET protocol. Participants completed questionnaires assessing posttraumatic stress symptoms (PTSS), general psychological distress, and trauma memory quality, wore a heart rate (HR) monitor assessing habituation, and were offered a Change Interview. At post-intervention, three participants showed reliable improvement in PTSS, but only one showed clinically significant change. One participant also demonstrated reliable improvement in general psychological distress. Effect size estimates ranged from moderate to very large and indicated change in the desired direction for all but one participant; estimated effects for general psychological distress were more modest. Three participants showed reductions in trauma memory quality, indicating increased integration. Within-session habituation was observed for all participants with available HR data; between-session habituation was also recorded for two of them. The lifeline was mentioned as a helpful aspect of NET, the video delivery was considered both a barrier and a facilitator to engagement, and positive or mixed changes were reported by two participants. Future research with more control and larger samples is needed to answer questions on generality of findings and impact of online delivery; future studies may also include longer follow-up periods and investigate other outcomes.

**Trial registration number** NCT04866511 (ClinicalTrials.gov).

## Introduction

### Impact of Witnessing Domestic Violence on Children and Young People

The term Domestic violence (DV) refers to a wide range of threatening behavior, violence or abuse between adults who are in a personal relationship (Howarth et al., 2013). It is estimated that 25% of children in the United Kingdom (UK) have been exposed to violence between adults in their home before the age of 18; of these percentage, 6% have witnessed severe DV, namely when the violence involved a high risk of significant harm or death (Radford et al., 2011).

It is well established that children and young people (CYP) who witness DV are susceptible to develop posttraumatic stress symptoms (PTSS; Levendosky et al., 2013), namely a pattern of reactions including intrusion symptoms connected to the experience, avoidance of associated stimuli, significant changes in mood and cognitions, and hyperarousal manifesting as hypervigilance, aggressive or reckless behavior (American Psychiatric Association, 2013). According to the Diagnostic and Statistical Manual of Mental Disorders, 5th Edition (DSM-5), posttraumatic stress disorder (PTSD) can be diagnosed if such symptoms persist for at least one month and cause significant impairment. Similarly, the International Classification of Diseases, 11th Revision (ICD-11) describes PTSD as characterized by re-experiencing, avoidance, and persistent perceptions of heightened current threat (World Health Organization, 2019). The PTSD diagnosis has been criticized for not adequately capturing the difficulties of survivors of repeated or chronic traumatization, which have often been described as *complex trauma* (Herman, 1992). In line with this, the ICD-11 introduced Complex PTSD as a distinct diagnosis with core PTSD symptoms and additional disturbances in affect regulation, self- concept, and relationships (Brewin, 2019).

Given its typical chronicity and repeated nature, exposure to DV can lead to complex trauma presentations; among CYP who witnessed violence in their homes, younger children are more likely to present with anxiety, including separation anxiety from the abused caregiver, physical symptoms and regression to previous stages of development, while older children and adolescents might externalize their distress by self- harming, misusing alcohol and substances, or physically attacking peers or other family members (UNICEF, 2016). Furthermore, CYP from violent households tend to perform poorly in school and are at greater risk of teenage pregnancy and offending behavior than those raised in homes without violence (Anda et al., 2001; Herrera & McCloskey, 2001). Some young people may also blame themselves for not protecting the abused caregiver (Kilpatrick & Williams, 1998). Finally, having witnessed DV in childhood may also increase the risk of entering a domestically abusive relationship in adulthood (Capaldi et al., 2012).

Both CYP and caregivers who have been exposed to DV should have access to adequate services that meet their diverse needs, including psychological therapies to address PTSS and other trauma-related difficulties (Lundy & Grossman, 2005). However, research in this area is still limited, possibly due to the recognition of the consequences of DV on children being a relatively recent phenomenon (Dodaj, 2020). Several trauma-focused therapies have shown good effectiveness with young people and are commonly offered in Child and Adolescent Mental Health Services (CAMHS) within the UK National Health Service (NHS). For example, Trauma-focused Cognitive Behavioral Therapy is considered the psychological treatment for traumatized youth with the strongest evidence base; however, such studies have not necessarily focused on young people with multiple or prolonged traumatic exposure (Smith et al., 2019) and dropout rates can be as high as 33% (de Arellano et al., 2014). Given the dramatic range of adverse outcomes for DV-exposed youth and the gaps in the evidence highlighted above, further research into effectiveness of therapies is paramount.

### Narrative Exposure Therapy and its Child-Friendly Adaptation

A treatment that has been specifically developed for people with multiple traumas is Narrative Exposure Therapy (NET; Schauer et al., 2011). NET is a short-term, manualized intervention combining Exposure Therapy and Testimonial Therapy that was initially conceived as a treatment for survivors of war and organized violence, and thus designed to be pragmatic and deliverable in low-resource and emergency settings. In NET, following assessment and psychoeducation, a chronological timeline of the client’s life (‘lifeline’) is constructed; positive (‘flowers’) and traumatic events (‘stones’), as well as losses (‘candles’) and any episodes of perpetrated violence (‘sticks’), are symbolically marked. The traumatic events are then narrated in detail, written up and read back to the client, and re-narrated until they have been processed through imaginal exposure. Finally, a written narrative of the client’s whole story is jointly produced and handed to them as a documented testimony.

NET has been included in the National Institute for Health and Care Excellence (NICE; 2018) clinical guidelines for the treatment of PTSD under the umbrella of trauma-focused CBT therapies. In a meta-analysis of NET studies on adults across different cultures (Lely et al., 2019), effect sizes for NET on PTSS were found to be large at post-treatment and sustained at follow-up, whereas medium effect sizes were found for depression at both points in time. Conversely, a different meta-analysis (Raghuraman et al., 2020), which also considered PTSD diagnostic status, found a significant reduction on this outcome following NET in the short- and midterm time points, which outperformed controls, but this was not maintained long term. Nonetheless, NET emerged superior to control interventions in terms of reduction of PTSS in the midterm and long-term. Furthermore, a review by Mørkved et al. (2014) suggested that, despite the many similarities, NET appeared to be more acceptable than Prolonged Exposure (PE), as reflected in its lower average dropout rates (PE: 27.20% vs. NET: 5.06%).

More recently, an adapted protocol (also known as the ‘KIDNET’) has been developed for use with CYP from age 7; differences from the adult version include involvement of caregivers in the psychoeducation session and use of visual and age- appropriate media and language to facilitate engagement and trauma narration (Schauer et al., 2011, 2017). Although fewer studies using this protocol have been published compared to the more established evidence for NET with adults, the available results in different trauma populations are promising (e.g., Catani et al., 2009; Hermenau et al., 2011; Onyut et al., 2005; Peltonen & Kangaslampi, 2019; Ruf et al., 2010).

To the authors’ knowledge, the only UK-based study conducted in a CAMHS setting is a feasibility report by Said and King (2020), which showed that NET helped reduce PTSS in unaccompanied asylum-seeking minors. Other applications of NET with children and adolescents in UK services include its use with young people with PTSD and psychotic symptoms, survivors of sexual abuse, with PTSD, anxiety, depression and eating difficulties, and with chronic health conditions requiring multiple interventions (Fazel et al., 2020). However, these examples are only available as narrative case reports and do not include quantitative outcomes. Furthermore, the impact of child-friendly NET on other mental health outcomes (aside from PTSS) is less conclusive (Siehl et al., 2021). As a brief, NICE-recommended treatment that was specifically designed to address multiple traumas and appears to be tolerable, NET may be a particularly good candidate for use with CYP who experience trauma-related difficulties following DV-exposure.

### NET Theoretical Background and Putative Mechanisms of Change

From a theoretical standpoint, NET draws upon dual representation theory (Brewin et al., 1996) and emotional processing theory (Foa & Kozak, 1986). In line with these perspectives, habituation and integration of traumatic memories into one’s general autobiographical memory have been proposed as key putative mechanisms that contribute to therapeutic change (Schauer et al., 2011). Habituation is achieved through repeated imaginal exposure to traumatic content, and it is reflected in a reduction in physiological arousal as the narration progresses. Some studies have found habituation to be linked to positive outcomes in exposure-based treatments (e.g., Craske et al., 2014). PTSS have been linked to lack of sufficient integration of trauma memories into autobiographical memory, which leads to such memories being fragmented, re-experienced at the sensory level rather than verbally retrieved, and triggered by environmental reminders (Brewin, 2014). Therefore, NET aims to relieve symptoms by helping the client contrast past and present sensory, cognitive, emotional and physiological experiences, and linking the arousal-dominated aspects of memory (‘hot memories’) to contextual information (‘cold memories’). This process gradually embeds the traumatic events within the context of the person’s whole life story (Neuner et al., 2008). Although there is preliminary evidence that NET can improve autobiographical integration by reducing trauma memory quality in traumatized young people (Kangaslampi & Peltonen, 2020; Isoaho et al., 2015), the mechanisms of change within this approach have been largely unexplored.

### Telepsychotherapy

In recent years, workgroups across different countries have also started to deliver NET remotely in response to the significant adaptations in healthcare service delivery during the COVID-19 pandemic (ARQ Centrum ’45, 2020). These changes have generally resulted in a renewed interest in telepsychotherapy, namely the use of real-time technologies, such as videoconferencing, to deliver psychotherapy to clients (Centers for Medicare and Medicaid Services, 2019).

Even in non-pandemic times, delivering psychological treatments remotely can have considerable advantages, including improving access to psychological interventions by reducing barriers such as travel distances and costs, conflicting schedules, and possibly stigma around being seen attending psychological services (Comer et al., 2014; Gloff et al., 2015). A systematic review and meta-analysis by Norwood et al. (2018) found video-delivered psychotherapy to be noninferior to face-to-face therapy in terms of target symptom reduction, however, the therapeutic alliance in video delivery was inferior to in-person delivery. With regard to trauma-focused therapies, a systematic review of studies comparing PTSD interventions for adult veterans delivered face-to-face versus by videoconference found that the two modalities were largely comparable in terms of outcomes, dropout rates, attendance rates, therapeutic alliance, and risk management (Turgoose et al., 2018).

Although the evidence supporting the use of telepsychotherapies for CYP is growing (e.g., Nelson & Sharp, 2016), the empirical literature on trauma-focused treatments for this group is still scarce. A recent meta-analysis (Venturo-Conerly et al., 2022) examined studies on remotely delivered psychotherapy with young people presenting with anxiety, depression, trauma, obsessive-compulsive, attention, and conduct conditions and problems; overall, pooled effect sizes at post-intervention and follow-up were similar to those found in meta-analyses of face-to-face youth psychotherapies. Factors associated with significantly larger effects included contact with the therapeutic provider (compared to fully computerized programs) and focus on anxiety and conduct problems. Additionally, an evaluation of Cognitive Behavioral Therapy delivered via videoconferencing in CAMHS (Porter et al., 2022) found that the majority of CYP in the sample significantly progressed toward their treatment goals and 31–38% showed reliable improvement in anxiety and depression symptoms; in this study, the video delivery was found to be acceptable and effect sizes were comparable to in-person delivery in routine practice settings. A meta-analysis (Fischer-Grote et al., 2024) of studies conducted with children, adolescents and young adults accessing remote and app-based mental health interventions after the onset of the Covid-19 pandemic showed similarly promising results (for example, effectiveness for depression, anxiety, and social functioning problems); however, none of the studies specifically investigated a trauma-focused intervention, no significant treatment effect was found for Covid-related trauma and other symptoms, and face-to-face delivery was a comparison in only one study, likely due to the reduced in-person provision during the pandemic. Examples of research on remotely delivered trauma-focused therapies for CYP include a small proof of concept feasibility study (Stewart et al., 2017) and an open pilot study looking at the feasibility and effectiveness of Trauma-focused Cognitive Behavioral Therapy for traumatized youth, which found preliminary support for both (Stewart et al., 2020).

To the authors’ knowledge, the only published examples of remotely delivered NET are a case report of an Afghani/Iranian refugee woman, who completed the therapy via telephone with the aid of an interpreter (Olavarrieta & Benuto, 2022), and a case study series of NET during pregnancy using partial video delivery (last two sessions only; Stevens et al., 2020). As the offer of remotely delivered therapies is likely to continue in parallel with face-to-face services, developing the evidence base in this area is crucial.

### Purpose and Objectives of Current Study

In light of the abovementioned gaps in the literature, the purpose of this study was to investigate the potential effectiveness, feasibility, acceptability and putative mechanisms of change of NET delivered via videoconferencing with CYP who witnessed DV. Specifically, the primary objective was to evaluate whether video NET can reduce PTSS in this client group. The secondary objectives were to: (i) determine whether video NET can also impact on general psychological distress, including depression symptoms, anxiety symptoms, risk to self, and functioning problems; (ii) examine the putative mechanisms of change within NET, i.e., habituation and integration of trauma memories; and (iii) explore whether video NET is feasible and acceptable.

## Methods

### Ethical Approvals

This study was reviewed and approved by the University of Nottingham Research Governance Team, a local Research Ethics Committee (REC), and the Health Research Authority within the NHS. The Research departments of two local NHS Trust also confirmed their capacity to support the study.

### Design

The study adopted a naturalistic Single Case Design (SCD) methodology, and specifically a mixed-method, AB, interventional time-series design. This was aimed to measure change at the level of individual participants in a case series by comparing their respective baseline, intervention, and follow-up (FU) phases.

### Recruitment and Consent Procedures

Young people open to Community CAMHS teams within two local NHS Trusts were considered eligible to take part in the study if they: (a) were aged 12–17 at the time of recruitment; (b) had witnessed DV in the past; (c ) were experiencing PTSS, as assessed by a CAMHS clinician; (d) were able to provide informed consent (if aged ≥ 16) or had a parent/guardian who could consent for them (if aged < 16); (e) were on the CAMHS waiting list and were not currently receiving another trauma-focused intervention; (f) were able to communicate verbally and speak English; and (g) had access to any device with a webcam, a mobile device, and Internet connection. Participants were excluded from the study if they had a diagnosis of Intellectual Disability, currently abused illicit substances, or had been assessed by a CAMHS clinician as being at high risk to themselves (e.g., severe non-suicidal self-injury [NSSI] and suicidality), risk from others (e.g., current exposure to DV or other forms of violence), and risk to others (e.g., serious and persistent violent behavior).

Participants were recruited between May and December 2021. Written consent and, where relevant, assent were received for all aspects of the study, including audio or video recording of sessions. If necessary, the CAMHS teams agreed to provide further and appropriate support to participants at the end of the study. Finally, young people and their parents were offered a pre-therapy call to arrange the initial NET session and discuss any questions or concerns, including around the use of the videoconferencing platform Microsoft Teams (MST).

### Measures

Information on participant demographics, history of trauma and adversity, main symptoms and presenting difficulties, any mental health and/or neurodevelopmental diagnoses, and previous interventions were retrieved from NHS electronic records.

Outcome measures, namely the Children’s Revised Impact of Event Scale – 13 (CRIES-13, Perrin et al., 2005) assessing PTSS, and the Young Person’s Clinical Outcomes in Routine Evaluation (YP-CORE; Twigg et al., 2009) assessing general psychological distress, were administered approximately weekly throughout the baseline, intervention (at the beginning of each session), and one-month FU phases. Ideally, the intervention phase in AB designs should commence once baseline stability has been reached (Morley, 2018). In this case, waiting for a stable baseline would have meant withholding the therapy from traumatized young people who were highly distressed, were experiencing lengthier than usual waiting times due to the Covid-19 pandemic, and who often struggle with engagement. Therefore, it was decided that participants would commence NET after three weeks, regardless of whether their baselines had stabilized or not.

The process measures assessing the two putative mechanisms of change were only collected during the intervention phase. The Trauma Memory Quality Questionnaire (TMQQ; Meiser-Stedman et al., 2007) was used to assess the problematic characteristics of traumatic memories resulting from a lack of autobiographical integration (e.g., fragmentation, sensory and ‘here-and-now’ quality, difficulty to put them into words, etc.). A wearable Heart rate (HR) monitor was used as a proxy measure of physiological arousal to assess: (a) within-session habituation (WSH) during narrative exposure; (b) and between- session habituation (BSH) as the narration progressed. Specifically, Huawei Band 4 Pro, a wearable activity tracker that includes a continuous HR monitoring function and can be connected to an app, was chosen for this study.

Participants completed all self-report measures via the online survey platform Jisc Online Surveys. At the end of FU, participants were asked to take part in a Change Interview (based on Elliott & Rodgers, 2008) via MST to assess their experience of taking part in video NET, any perceived changes, and any suggestions for improvement. To reduce response bias, interviews were conducted by an independent researcher, namely a Trainee Clinical Psychologist who was not involved in either the NET sessions or study development. Table 1 displays an overview of description, administration schedule, and psychometric properties of all measures.

**Table 1:** Description, Administration schedule, and Psychometric properties of the study measures

Measure	Variable	Description and scoring details	Administration schedule	Psychometric properties
CRIES-13	Primary outcome: posttraumatic stress symptoms	13 items assessing symptoms over the previous week; 4-point Likert scale (from 0 = “Not all” to 5 = “Often”). Subscales: intrusion, avoidance, and hyperarousal symptoms. All items are added up to produce a total PTSS score and subscales are added up to produce cluster-specific scores; higher scores indicate higher levels of PTSS. Clinical cut-off is 30.	Baseline (weekly, x 3) Intervention (each session) Follow-up (weekly, x 3)	High internal consistency, and concurrent validity (Giannopoulou et al., 2006). Satisfactory construct validity (Giannopoulou et al., 2006; Pereira et al., 2021). Good test-rested reliability (Deeba et al., 2014).
YP-CORE	Secondary outcome: general psychological distress	10 items assessing general psychological distress, including anxiety symptoms, depression symptoms, risk to self, and functioning, over the past week. 5-point Likert scale (from 0 = “Not at all” to 4 = “Most or all of the time”). Functioning items are reverse scored. All items are added up to produce a total distress score; higher scores indicate higher levels of distress. Clinical cut-off: 10.3–15.9, depending on age and gender.	Baseline (weekly, x 3) Intervention (each session) Follow-up (weekly, x 3)	High internal consistency (O’Reilly et al., 2016; Twigg et al., 2016). Good sensitivity to change (Twigg et al., 2009, 2016). Good reliability, validity, and acceptability (Twigg et al., 2009).
TMQQ	Process variable: traumatic memory quality as an indicator of poor autobiographical integration	11 items assessing properties of traumatic memories. 4-point Likert scale (from 1 = “Don’t agree at all” to 4 = “Completely agree”). Item 6 (“I can talk about what happened very easily”) is reverse scored. All items are added up to produce total score; higher scores indicate higher trauma memory quality and poorer integration.	Intervention (each session from session one)	Good internal consistency, criterion validity, and construct validity (Meiser-Stedman et al., 2007).
Huawei Band 4 Pro	Process variable: physiological arousal as indicator of habituation	Wearable activity tracker (wristband) with continuous HR monitoring function. Measures Beats per minute (BPM). Can be connected to manufacturer’s app (Huawei Health).	Intervention (each session from session three).	-
Change Interview	Feasibility and acceptability	Interview protocol exploring participants’ experiences of the intervention and perceived changes.	Approximately one-month follow-up.	-

Notes. CRIES-13 = Children’s Revised Impact of Events Scale-13 items; YP-CORE = Young Person’s Clinical Outcomes in Routine Evaluation; TMQQ = Trauma Memory Quality Questionnaire

### Video NET Intervention

The intervention followed the KIDNET protocol and comprised six to ten sessions lasting between 90 and 120 min. The exact intervention length for each participant was contracted on a case-by-case basis depending on clinical need, including the number of traumatic events each young person experienced and wished to discuss in treatment. As per study protocol, participants who completed at least the minimum number of contracted sessions (six) were considered treatment completers. Although NET has been found to reduce PTSS in adolescents and young adults after only four sessions (Schaal et al., 2009), a minimum intervention ‘dose’ of six was chosen to allow for dedicating a minimum of four sessions to processing of traumatic events. Due to the changes in service provision during the Covid-19 pandemic and the scope of the study, sessions were delivered remotely via MST. The delivery followed the guidelines on conducting online NET safely and effectively; these include discussion and use of grounding strategies that can be facilitated remotely to prevent dissociation and manage distress, as well as considerations around confidentiality, technical set-up, and management of disruptions (Kaltenbach et al., 2021b). These guidelines also state that remotely delivered NET sessions should mirror face-to-face ones as far as possible in terms of structure, and do not propose any specific adaptations with regard to treatment length. This is consistent with the literature on adaptations to remote provision in UK psychological therapy services during COVID-19, which showed that the number of sessions offered and attended remained consistent with previous, in-person provision (Capobianco et al., 2023).

All sessions were delivered by the lead researcher and therapist (FR), a Trainee Clinical Psychologist with training in and experience of using NET in clinical practice, and took place between June 2021 and February 2022. Treatment adherence and competence were assessed by another researcher (TS) with extensive experience of the model and were aided by the session recordings and quality check forms, which were based on the treatment manual and KIDNET literature (Schauer et al., 2011, 2017), and the abovementioned guidance around online NET. A full breakdown of the sessions content is presented in Table 2.

**Table 2:** Breakdown of content of video NET sessions

Session	Activity
1	• Review of symptoms and difficulties (questionnaires and discussion); • Psychoeducation^a^ including normalization and validation of common posttraumatic symptoms and responses, explanation of ‘hot’ and ‘cold’ memories, and how NET/KIDNET works and looks like. Optional presence of nonviolent parent/guardian; • Safety planning: additional participant contact (telephone), emergency contacts, strategies to manage dissociation and distress.
2	• Review of symptoms and difficulties (questionnaires and discussion); • Construction of Lifeline.
3	• Review of symptoms and difficulties (questionnaires and discussion); • Narrative exposure: start of detailed narration from birth, including the first traumatic event (stone) on the lifeline; • End of narration at a point of (relative) safety; • Grounding and re-orientation to the present, if needed.
4–9 (or up to any penultimate session)	• Review of symptoms and difficulties (questionnaires and discussion); • Re-narration: therapist reads written summary of previous event(s) to young person. New details are added and therapist assesses signs of habituation; • Narrative exposure process is repeated for the main events that are causing distress to the young person, in chronological order; • Less significant traumatic/negative events, losses, and positive events are discussed briefly and included in the story. Some flowers (positive events) may be narrated in detail if appropriate.
10 (or any final session)	• Review of symptoms and difficulties (questionnaires and discussion); • Therapist and young person review the written narrative and finalize the story; • Construction of second lifeline (optional) and focus on the future, including young person’s hopes and goals; • Therapist sends the written narrative to the young person via protected email or post.

Note. ^a^Psychoeducation handouts shared with young person via the ‘screen share’ function in MST and then emailed after the session

All therapy and study materials were posted to participants before commencing the sessions. At the end of therapy, participants were debriefed and the lead researcher/therapist liaised with CAMHS clinicians to discuss post-NET support as appropriate.

### Analyses

Visual analysis (Lane & Gast, 2014) was used to systematically examine changes within and across phases in the outcome measure and one of the process measures (TMQQ). Tau-U indices (Parker et al., 2011) were chosen to strengthen the visual analysis and estimate effect sizes for video NET on the two outcome measures, whilst accounting for potential baseline trends. Any observed trends in the baselines were first tested for statistical significance through the free online calculator by Tarlow (2016). If baseline trends were found to be nonsignificant, the Tau-UA vs. B (i.e., without baseline trend adjustment) was calculated at post-treatment (final session), one-week, and one-month FU through an effect size web application in RStudio (Pustejovsky et al., 2021). The absolute values of TAU-U indices were interpreted as follows: (a) very large: > 0.80, large: 0.60 − 0.80, moderate: 0.20 − 0.60, small: <0.20 (Vannest & Ninci, 2015); and (b) indices with a minus sign indicated effects in the desired direction (i.e., symptom reduction). Missing data were handled by imputing the mean of the available scores in the same phase, a common imputation method in SCDs (Velicer & Colby, 2005).

The Reliable Change and Clinically Significant Change ([RC] and [CSC]; Jacobson & Truax, 1991) methods were be applied at post-treatment and FU for both outcome measures, to determine whether any changes were reliable (i.e., beyond chance or measurement error at 95% confidence) and clinically significant (i.e., indicating non-clinical levels of PTSS and general psychological distress). Additionally, Simulation Modelling Analysis (SMA; Borckardt et al., 2008) was used to investigate temporal relationships between the CRIES-13 and the YP-CORE (i.e., whether changes in PTSS were followed by changes in general psychological distress).

The HR data recorded by the Huawei Band 4 Pro during the narrations of traumatic events were graphed and visually inspected. Specifically, HR readings at ‘peak arousal’ and end of session were compared to investigate WSH and to peaks in the following sessions to examine BSH.

Finally, qualitative findings from the Change Interviews were analyzed through content analysis using a deductive approach (Kondracki et al., 2002), and integrated with the quantitative data through a concurrent triangulation design (Creswell et al., 2003) in the discussion section.

## Results

### Participants and Intervention Delivery

A total of seven young people who met the eligibility criteria were approached about the study and five female participants aged 13–17 years (*M* = 15.4, *SD* = 1.82) were recruited from one of the Community CAMHS teams. Participant demographic and clinical information is detailed in Table 3; pseudonyms were chosen by the participants themselves and used throughout to protect their identities. All participants had witnessed multiple incidents of DV, as well as experienced additional traumatic events and/or adversities. All lived with the non-violent parent or a legal guardian at the time of the study. They all showed complex clinical presentations, including PTSS and a range of additional symptoms or functioning difficulties, including regarding school attendance.

**Table 3:** Participant demographic and clinical information

Information	‘Kaitlin’	‘Willow’	‘Nell’	‘Lilly’	‘Gem’
Age	17	13	17	14	16
Gender	Female	Female	Female	Female	Female
Ethnicity	White British	White British	Other ethnic background (Arab)	White British	White British
Brief description of witnessed DV	Up to age 4, from father to mother Aged 11–12, from stepfather to mother	Up to age 5, from father to mother	Up to age 5/6, from father to mother	Up to age 7/8, from father to mother	Aged 7–13, from father to mother
Main presenting difficulties	PTSS Social anxiety Depression symptoms Eating difficulties School difficulties	PTSS Separation anxiety Behavioral symptoms NSSI School difficulties	PTSS Depression symptoms Voice hearing/auditory hallucinations NSSI Dissociative symptoms School difficulties	PTSS OCD symptoms Behavioral symptoms Voice hearing/auditory hallucinations NSSI Dissociative symptoms School difficulties	PTSS Depressive symptoms Voice hearing/auditory hallucinations NSSI Suicidal ideation School difficulties
Diagnosis	ARFID (historical) Dyslexia	ASD	ASD BPD (provisional)	Possible ASD Dyslexia	-

Note. ARFID = Avoidant/restrictive food intake disorder; ASD = Autistic spectrum disorder; BPD = Borderline personality disorder; DV = Domestic violence; NSSI = Non-suicidal self-injury; PTSS = Posttraumatic stress symptoms; All names used to identify participants are pseudonyms

Although NET does not ordinarily require a formal symptom stabilization phase (Schauer et al., 2011; 2020), all participants had received previous therapeutic input, including at the individual and family level. The families of those who reported NSSI and/or suicidal ideation had been previously given safety plans by CAMHS clinicians. Although the referring CAMHS clinicians received regular updates, only ‘Willow’ had regular care-coordination meetings (mainly focusing on school difficulties) whilst accessing video NET.

Four out of five participants completed at least six sessions (the minimum intervention ‘dose’); the number of completed sessions ranged between four (‘Lilly’) and ten (‘Willow’). However, one participant (‘Gem’) was withdrawn from the study after session six due to an escalation in risk, namely an overdose with reported suicidal intent. Although Gem did not mention the intervention among the triggering factors and the attempt did not immediately follow the session, this was reported to the REC in keeping with the study protocol. No other adverse events, such as NSSI or acute inpatient admissions, occurred during the study.

Given that the number of stones (i.e., traumatic/negative events) participants included in their lifelines greatly exceeded the available sessions, the therapist and each participant jointly selected the stones that were associated with the highest level of distress in the present and focused on those during the narrative exposure process. Nonetheless, the other stones were acknowledged, summarized, and included in the written narrative. The intervention delivery was adapted to meet participants’ specific needs and overall was found to satisfy the established quality checks of adherence and competence.

### Potential Effectiveness for PTSS and General Psychological Distress

**Fig. 1:**
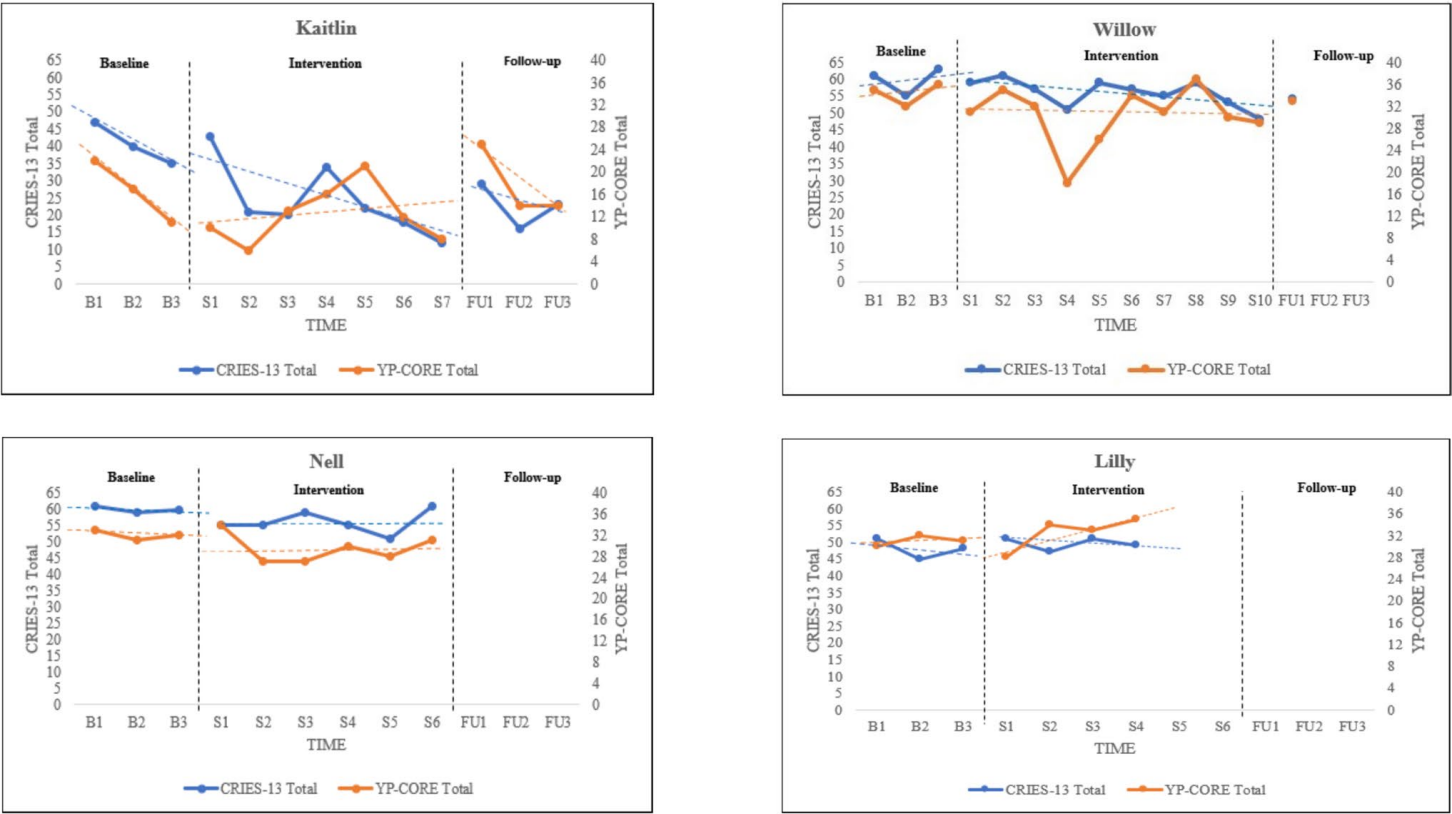
Visual Displays of Posttraumatic Stress and General Psychological Distress Symptoms for each Participant. Notes. B = Baseline; S = Session; FU = Follow-up; CRIES-13 = Children’s Revised Impact of Event Scale – 13; YP-CORE = Young person’s Clinical Outcomes in Routine Evaluation. Session measures were collected at the beginning of each session

Visual displays of participants’ CRIES-13 and YP-CORE scores are presented in Fig. 1. Within-phase linear trends are described below; for across-phase comparisons, see section on TAU-U A vs. B effect size estimates. In terms of PTSS, baseline CRIES-13 total scores revealed varying degrees of improvement, namely symptom decrease, for Kaitlin, Nell and Lilly; conversely, Willow and Gem demonstrated deterioration, namely symptom increase, during the baseline period. As Nell and Lilly did not complete the outcome measures on the last baseline point, these data were imputed by calculating the respective baseline means. Kaitlin and Willow demonstrated a downward trend, indicating improvement, across the intervention and FU phases, whilst Lilly and Gem showed improvement (albeit to different degrees) in the intervention phase but did not complete any FU measures. Only Nell’s scores revealed no trend, indicating symptom stability, during the intervention and, again, no FU data were available. Missing data during FU were not imputed. For Kaitlin and Willow, peaks in PTSS at the start of FU were noted to coincide with external life stressors (e.g., school-related events).

With regard to general psychological distress, as assessed by the YP-CORE, Kaitlin, Nell, and Lilly were found to be improving during the baseline phase, whilst Willow and Gem were deteriorating. Kaitlin and Willow, who completed measures during both intervention and FU, demonstrated a slight upward trend (indicating deterioration) and no trend, respectively, across those phases. A trough in Willow’s score at session four, indicating improvement, was noted to coincide with the achievement of personally meaningful goals in the previous week. For those who did not complete FU measures, trends in the intervention phase indicated the following: little to no improvement for Nell, slight deterioration for Lilly, and improvement for Gem. Missing YP-CORE data in the baseline and FU were handled as per the CRIES-13.

When analyzing temporal relationships between the CRIES-13 and the YP- CORE scores, SMA showed significant correlations for the following participants: Kaitlin at Lag + 2 (*r* = – .46, *p* = .038), Willow at Lag 0 (*r* = .60, *p* = .013), and Gem at Lag − 1 (*r* = .75, *p* = .012). These suggested that CRIES-13 and YP-CORE scores changed concurrently for Willow and sequentially for Kaitlin and Gem. Specifically, for Kaitlin, decreases in PTSS were associated with increases in general distress two weeks later; for Willow, decreases in PTSS were associated with decreases in general distress in the same week; and for Gem, decreases in PTSS were linked to decreases in general distress in the previous week.

When estimating effect sizes for video NET, Tau-UA vs. B was calculated for all participants on both outcome measures, as none of the baseline trends were found to be statistically significant at *p* < .05. Point estimates of effect sizes for PTSS (Table 4) ranged between moderate and very large at post-intervention, and for all but one participant (Lilly), they indicated presence of effect in the desired direction. For participants with available FU data, effect size estimates (calculated as intervention phase + FU points) were moderate to very large at one-week FU and very large at one-month. Effect estimates were found to be statistically significant (i.e., 95% CIs did not include 0) for Gem at post-intervention and for Kaitlin at one-week and one-month FU.

**Table 4:** Tests for Trend significance and effect sizes for posttraumatic stress symptoms

Participant	Baseline trend τ	Baseline trend *p*	Post-treatment Tau-UA vs. B [CI] (S#)	One-week FU Tau- UA vs. B [CI]	One-month FU TAU-UA vs. B [CI]
‘Kaitlin’	–1.00	0.296	–0.81 [–0.98, 0.01] (S7)	–0.83* [–0.98, –0.03]	–0.87* [–0.99, –0.10]
‘Willow’	0.333	1.000	–0.53 [–0.88, 0.22] (S10)	–0.58[–0.89, 0.17]	-
‘Nell’	–0.333†	1.000	–0.67†[–0.94, 0.16] (S6)	- -	- -
‘Lilly’	–0.333†	1.000	0.33† [–0.47, 0.83] (S4)	- -	- -
‘Gem’	0.816	0.540	–1.00* [–1.00, − 1.00] (S6)	-	-

*Notes.* Tau-UA vs. B = Tau-U with no baseline trend correction; S# = Session number; CI = Confidence interval at 95%; * = statistically significant at *p* < .05

† = Calculated with mean of baseline imputation method; - = effect size not calculated due to missing FU data; effect size with a negative sign indicate effect in the desired direction (symptom reduction); effect size interpretation: very large: > .80, large: .60 - .80, moderate: .20 - .60, small: <.20.

For general psychological distress (Table 5), post-intervention estimated effect sizes were moderate for four participants and very large for one (Gem), and indicated effect in the direction of clinical improvement for all but Lilly. For Kaitlin and Willow, point estimates remained moderate and in the desired direction at one-week FU, and a moderate effect was also found for Kaitlin at one-month FU. Finally, Gem’s effect size was found to be statistically significant (i.e., 95% CI did not include 0) at post-intervention.

**Table 5:** Tests for Trend significance and effect sizes for General Psychological Distress

Participant	Baseline trend τ	Baseline trend *p*	Post-treatment Tau-UA vs. B [CI] (S#)	One-week FU Tau- UA vs. B [CI]	One-month FU TAU-UA vs. B [CI]
‘Kaitlin’	–1.000	0.296	–0.52 [–0.88, 0.26] (S7)	–0.33 [–0.79, 0.39]	–0.33 [–0.78, 0.37]
‘Willow’	0.333	1.000	–0.60 [–0.90, 0.16] (S10)	–0.58 [–0.89, 0.17]	-
‘Nell’	–0.333†	1.000	–0.61† [–0.92, 0.21] (S6)	- -	- -
‘Lilly’	0.333†	1.000	0.50†[–0.36, 0.89]	- -	-
‘Gem’	–0.816	0.540	–0.89* [–0.99, –0.05] (S6)	-	-

*Notes.* Tau-UA vs. B = Tau-U with no baseline trend correction; S# = Session number; CI = Confidence interval at 95%; * = statistically significant at *p* < .05

† = Calculated with mean of baseline imputation method; - = effect size not calculated due to missing FU data; effect size with a negative sign indicate effect in the desired direction (symptom reduction); effect size interpretation: very large: > .80, large: .60 - .80, moderate: .20 - .60, small: <.20.

All participants scored within the clinical range at pre-intervention (first baseline observation) on the CRIES-13 and the YP-CORE. With regard to RC and CSC analyses, three out of five participants demonstrated reliable improvement on the CRIES-13 at post- intervention. This was maintained at one-week and one-month FU for Kaitlin, who also showed clinically significant improvement at both points. The remaining four participants remained in the clinical range. Kaitlin was also the only participant to record reliable and clinically significant improvement at post-intervention on the YP-CORE; this was lost at one-week FU and regained at one-month. No participant showed reliable deterioration. An overview of the results of the RC and CSC analyses is presented in Table 6.

**Table 6:** Reliable Change and clinically significant change on the outcome measures

Participant	Time	CRIES-13	YP-CORE
‘Kaitlin’	Pre (B1)	47	22
	Post (S7)	12R + C+	8R + C+
	One-week FU	29R + C+	25
	One-month FU	23R + C+	14R + C+
‘Willow’	Pre (B1)	61	35
	Post (S10)	48R+	29
	One-week FU	54	33
	One-month FU	-	-
‘Nell’	Pre (B1)	61	33
	Post (S6)	61	31
	One-week FU	-	-
	One-month FU	-	-
‘Lilly’	Pre (B1)	51	30
	Post (S4)	49	35
	One-week FU	-	-
	One-month FU	-	
‘Gem’	Pre (B1)	53	35
	Post (S6)	39R+	33
	One-week FU	-	-
	One-month FU	-	-

*Notes*. RC and CSC calculated using clinical and non-clinical norms from published literature; ^R+^ = Reliable improvement (relative to first baseline score); ^C+^ = Clinically significant improvement (external criterion/criterion C for clinical cut-off); - =not available due to missing FU data

### Putative Mechanisms of Change

**Fig. 2:**
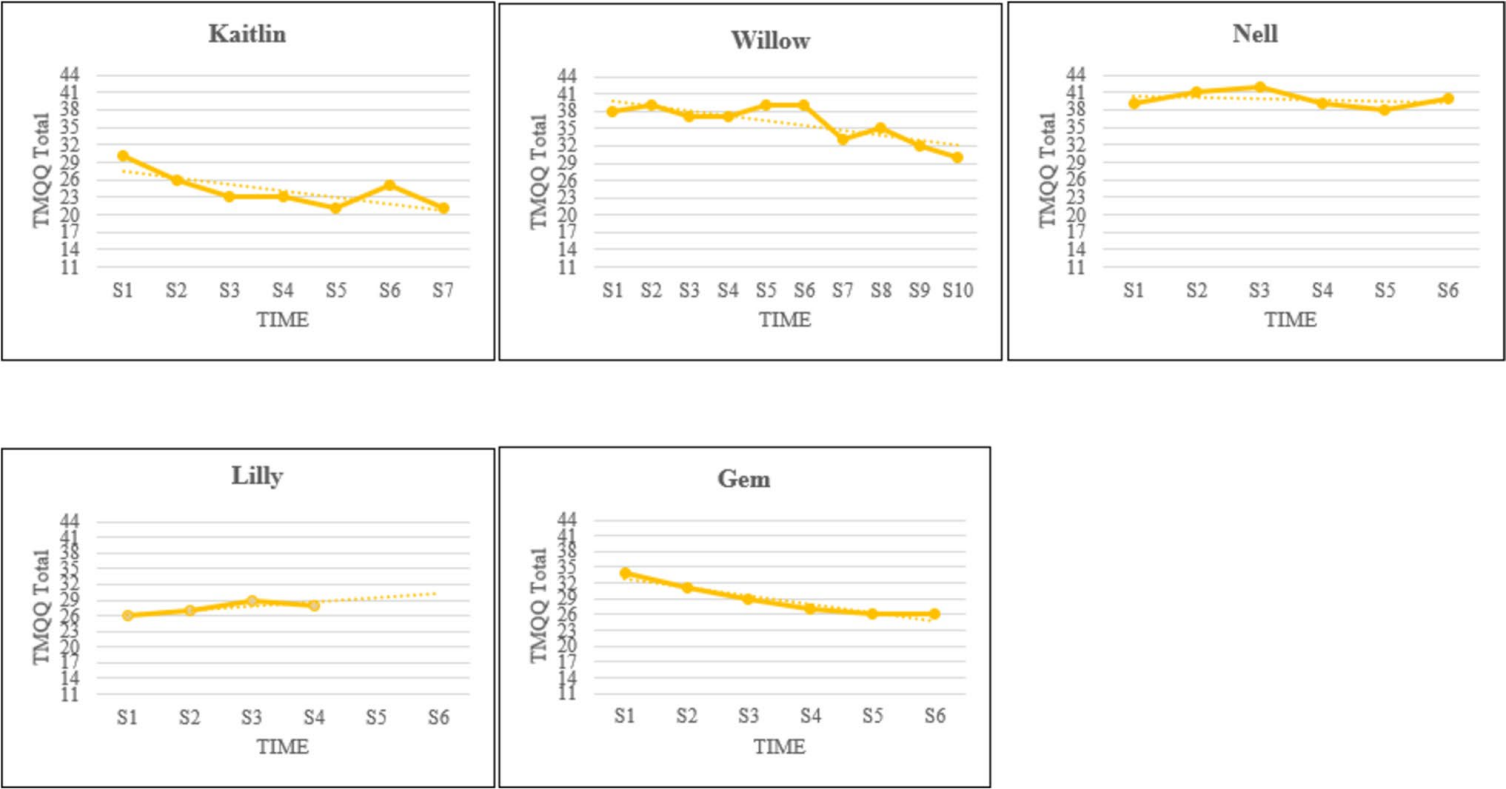
Visual Displays of Trauma Memory Quality during the Intervention Phase for each Participant. Note. S = Session; TMQQ = Trauma Memory Quality Questionnaire; Lower scores indicate decreased trauma memory quality and increased integration

Visual analyses of TMQQ data showed that trauma memory quality decreased during the intervention phase for three out of five participants, suggesting improvements in memory integration. Specifically, for these participants the TMQQ total score decreased by 8/9 points between the first and the last session. Conversely, little to no change, as shown by the lack of trend, was observed for Nell and a slight deterioration (upward trend) was recorded for Lilly. Visual displays of TMQQ scores are presented in Fig. 2.

**Fig. 3:**
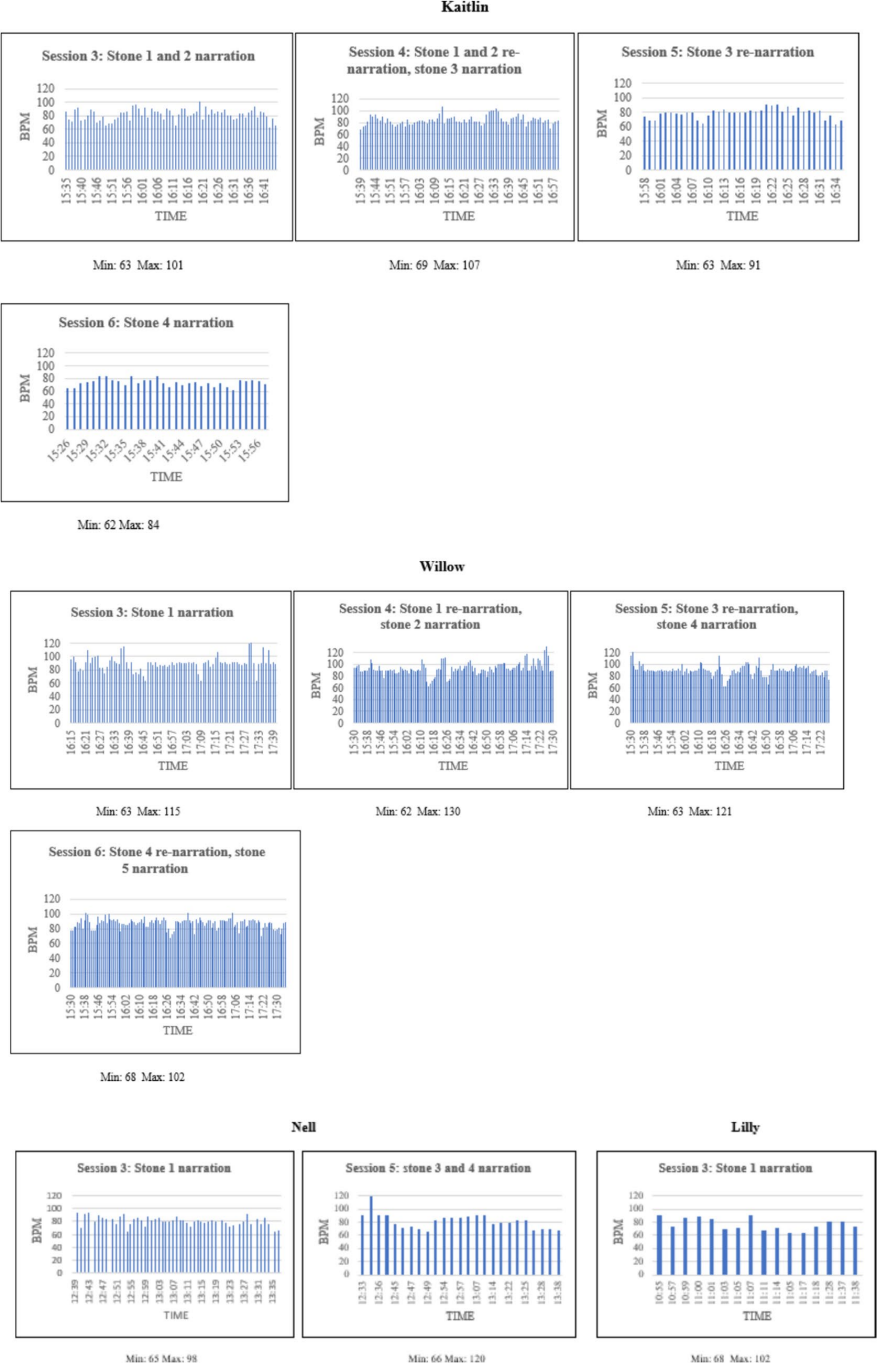
Heart Rate Recordings during Narrative Exposure Sessions for Four Participants. Note. BPM = Beats per minute

HR data during narrative exposure, starting from session three, were available for four out of five participants. HR readings were not available for some sessions due to technical issues or participants not wearing the devices. Overall, participants showed varying degrees of habituation. WSH was observed for all participants, as suggested by an increase in physiological arousal during narration of stones, followed by a decrease at the end of the session (or narration of each stone, in the case of two stones being narrated in one session). BSH, as shown by decrease in ‘peak arousal’ (maximum BPM value) across sessions, was observed for Kaitlin and Willow, although for both peak arousal increased between session three and four before declining in the following sessions. BSH was not observed for Nell and could not be established for Lilly, as HR readings were only available for one session. Figure 3 shows the visual displays of available HR readings for four participants.

### Change Interviews

Four out of five participants agreed to take part in the Change Interviews at the end of FU, however, only Kaitlin and Lilly actually completed them. One participant (Willow) cited not wanting to speak with an unknown researcher as the reason for later declining the interview. With regard to their overall experience of video NET, Kaitlin described the therapy as challenging but acceptable, whilst Lilly commented that, although she could see how it could be beneficial, she did not think it was the right fit for her. Both commented positively on the psychoeducation and lifeline sessions, with Kaitlin particularly appreciating the visual medium of the lifeline and Lilly valuing the inclusion of positive events. The structure of the sessions and overall therapy was seen as both helpful and excessively repetitive, and both participants described some level of ambivalence around narrating their traumatic experiences in detail. Kaitlin and Lilly reported opposite experiences of the video delivery aspect: the former shared that doing NET via videoconferencing was less intimidating and enabled her to engage; the latter identified significant barriers, such as losing concentration more easily, having the sessions in the same environment where the DV took place, and struggling to develop a relationship with the therapist. Changes related to the therapy included improvements in mood and relationships with peers, as reported by Lilly, whilst Kaitlin described increased emotional openness but also greater irritability. The narrative approach and being specifically asked about feelings were highlighted as helpful aspects of NET, and the session length was mentioned as an unhelpful aspect.

## Discussion

This study primarily aimed to investigate whether the child-friendly NET protocol, delivered via videoconferencing, can reduce PTSS in children and young people who witnessed DV and accessed mental health services in the UK. Secondary aims were to explore the potential impact of video NET on general psychological distress, investigate its putative mechanisms of change, and assess its feasibility and acceptability.

### Clinical Effectiveness

Taken together, the findings on the impact of video NET on PTSS showed that three participants (Kaitlin, Willow, and Gem) out of five demonstrated improvement at post- intervention (downward trend, reliable improvement, and moderate to very large effect sizes in the desired direction), and two showed a mixed picture (Nell: no trend, no reliable change, and moderate effect size in the desired direction; Lilly: downward trend, no reliable change, and moderate effect size in the opposite direction).

Although only one participant also reported clinically significant change, these findings provide preliminary evidence for the effectiveness of video NET to reduce PTSS, in line with SCD standards literature stating that at least three demonstrations of effect are required to draw conclusions about treatment effectiveness (e.g., Lobo et al., 2017). The results are also consistent with the existing literature on NET face- to-face delivery and use with youth who experienced other types of traumatic events across different cultural contexts (Siehl et al., 2021), including those who experienced both refugeedoom and DV (Peltonen & Kangaslampi, 2019).

It should be noted that two out of the three participants who appeared to benefit from the intervention were older adolescents; similarly, a meta-analysis by Lely et al. (2019) found an age-related effect for NET, suggesting that the older the participants, the more effective NET was. This is not surprising, considering that therapeutic process in NET involves reflection on and meaning-making about one’s life history, including insight into personal experiences and perpetrators’ motivations, which is typically more challenging for children and younger adolescents (Schauer et al., 2017).

With regard to whether the effects of video NET were sustained, this was difficult to establish due to the amount of missing data in the FU phase. The two participants with available FU data showed either evidence of sustained therapeutic gains (very large effects that were reliable and clinically significant) at one-week and one-month FU or possible sustained change (moderate effect but no reliable change) after one week. Albeit limited, these findings partially align with those of a recent meta-analysis on NET long-term efficacy, which found that therapeutic gains for NET increase over time, particularly when assessed at ≥ 6-month FU; the authors hypothesized that this is due to trauma processing continuing after the end of NET and extending to similar events, in keeping with the theoretical assumptions of fear networks (Siehl et al., 2021). In light of this, it is possible that the gains associated video NET would have become more evident if assessed after a longer time period.

Overall, the present findings on general psychological distress appear to mirror those for PTSS in terms of number of participants who showed some level of improvement or little to no change. However, the effect size estimates were generally more modest and only one participant demonstrated reliable change and scored within the non-clinical range at post-intervention and one-month FU. This is consistent with the literature on face-to-face NET with adults, which showed that NET is generally effective to a lesser degree on secondary outcomes such as depression (Lely et al., 2019; Siehl et al., 2021). The findings are also in line with some of the evidence for the KIDNET protocol for secondary outcomes, for example, RCTs and cohort studies showing small to moderate effects for depression at post- intervention and FU (e.g., Hemenau et al., 2011; Park et al., 2020). Conversely, other studies have shown more promising results, such as an 100% remission rate for depression symptoms (Onyut et al., 2005), and significant reductions in functioning problems (Catani et al., 2009).

### Integration of Traumatic Memories and Habituation

This study found a decline in trauma memory quality as assessed by the TMQQ in three out of five participants, indicating that their traumatic memories were less sensory-based and incoherent and more verbally available at the end of the intervention. These participants also reported a similar degree of change when comparing scores between the first and the last session. The other two participants showed either no change or a slight increase in trauma memory quality. Overall, these findings for one of NET putative mechanisms of change are in line with those for the primary outcome, as participants who described their traumatic memories as more integrated were also those who showed reliable changes in PTSS at post- intervention. The reverse was true for those who did not report improvements in memory quality. This suggests a link between process (trauma memory integration) and outcome (PTSS) for young people in this sample. Similarly, Kangaslampi and Peltonen (2020) found a significant improvement in memory quality, as well as a significant correlation between improvements in traumatic memories and symptom reduction, in young people who received the child-friendly NET protocol through face-to-face delivery; however, contrary to the authors’ expectations, KIDNET did not affect trauma memory quality to a greater extent than treatment as usual.

With regard to habituation, the findings are somewhat limited by the amount of missing HR recordings. However, the observed WSH during narrative exposure sessions for all four participants with available data is consistent with the theoretical underpinnings of NET, which suggest that this approach fosters habituation through its characteristic ‘exposure conversation’ style (Robjant et al., 2017). Similarly to what stated above for trauma memory quality, BSH was observed for two out the three participants who showed reliable PTSS changes at post-intervention (Kaitlin and Willow), whilst no HR data were available for the third one (Gem). Again, this tentatively suggests a link between process (habituation following exposure to traumatic content) and outcome (PTSS) in the present study, although this only true for habituation across the therapy sessions.

The above findings reflect the mixed evidence for the notion that WSH and BSH are both predictive of treatment outcome in exposure-based interventions, which has emerged from studies with adults (e.g., Craske et al., 2014; Rauch et al., 2004). Conversely, Peterman et al. (2019) found that neither WSH nor BSH predicted treatment outcomes in young people with anxiety disorders who received CBT. Overall, the findings on habituation during video NET in the present study align with those from a review of PE mediators, which highlighted BSH among the variables with strong evidence and WSH as one with limited support (Cooper et al., 2017).

### Feasibility and Acceptability

Qualitative participant reports provided tentative evidence for the feasibility and acceptability of video NET for DV-affected young people in this sample, especially since the views of three participants were not represented. The dropout rate for video NET (20%) was higher than that the figures reported in other KIDNET literature (0–12%; Catani et al., 2009; Onyut et al., 2005; Ruf et al., 2010). However, the interview of the young person who dropped out before session six (Lilly) suggested that intervention discontinuation was due to factors associated with the video delivery and the perceived lack of fit between the therapy and her current needs, rather than to lack of acceptability of the protocol per se.

Although the low interview uptake may in itself raise questions about acceptability, both participants who completed the interviews shared that video NET was or could be beneficial, highlighted helpful and unhelpful aspects, and reported positive or mixed changes associated with the intervention. In particular, the reported appreciation for the lifeline exercise and inclusion of flowers (i.e., positive events and resources) is consistent with a published case example of NET use with a male adolescent, who reflected that completing the lifeline as the first task facilitated his engagement in treatment (Fazel et al., 2020). Other qualitative findings from the present study, such as participants’ ambivalence towards discussing their traumatic experiences, the importance of the narrative approach, and the usefulness of psychoeducation, broadly echo some of the themes emerging from Said et al. (2021), who explored unaccompanied asylum-seeking minors’ experiences of receiving NET within a UK CAMHS Team.

The use of videoconferencing to deliver NET was highlighted by participants as both a barrier and a facilitator to engagement. A similar ambivalence was captured by Worsley et al. (2022) in their qualitative study of CYP’s, parents’, and clinicians’ experiences of remote therapy in CAMHS. In particular, young people described increased accessibility and flexibility, feeling safer and more comfortable in their home environments, and feeling more in control during interactions with the therapist as advantages of online therapy. Conversely, some young people reported disadvantages such as exposure to distractions that affected their concentration, privacy concerns, and negative impact on the therapeutic relationship. Similarly, in Mekori-Domachevsky et al. (2021), adolescents and caregivers accessing a mental health outpatient clinic in Israel reported an overall satisfaction with online service provision during the pandemic; however, young people’s higher levels of internalizing symptoms were found to negatively correlate with therapeutic alliance scores, suggesting that anxious and depressed adolescents might particularly struggle to build relationships with clinicians in online settings.

### Strengths and Clinical Implications

To the authors’ knowledge, this is the first NET study to focus exclusively on young people who witnessed DV and to be conducted in UK CAMH services with a non-refugee population. It is also the first completed study to investigate the exclusive videoconferencing delivery of a NET protocol, although the authors are aware of ongoing projects (e.g., Kaltenbach et al., 2021a study protocol). Furthermore, this is one of the few studies employing the KIDNET protocol to explore both clinical outcomes and the two main putative mechanisms of change within this therapeutic approach.

Albeit preliminary, the findings of this study add to the evidence base for KIDNET and that for complex trauma interventions more broadly. The most effective treatments for complex trauma presentations are the subject of ongoing debates and current guidelines on their use with young people highlight the need for further research, as they acknowledge that those with more severe clinical presentations tend to benefit less from standard evidence-based treatments (Berliner et al., 2019).

As a brief trauma-focused intervention with promising outcomes, video NET may be a valuable addition to the existing CAMHS provision of treatments to support traumatized youth to process their traumatic experiences. Its time-limitedness may be particularly advantageous in light of the high demands and resource constraints that healthcare services are currently facing. Furthermore, there is some evidence that the skills to deliver NET protocols can be successfully disseminated within services through relatively brief training and supervision of less experienced clinicians (e.g., Köbach et al., 2017). Both these benefits are particularly important as referrals of DV-affected young people may be increasing, a consequence of the preliminary evidence of rising rates of DV during the Covid-19 pandemic (Bradbury- Jones & Isham, 2020).

Some participants in the current sample also necessitated further input after completing video NET. It is possible that, for young people with such complex presentations and needs as those in this study, video NET may only be a component of a broader care plan. When traumatic experiences occur within the context of primary attachment relationships, only subduing fear-based responses is often insufficient (Courtois & Ford, 2015). Although NET gives patients space to process significant losses, young people who witnessed DV may need additional time and support to come to terms with losing or feeling betrayed by an attachment figure, as well as to regulate their often complex emotions toward the violent caregiver (Ford & Courtois, 2013). Additionally, a proportion of the young people in this sample had either diagnosed or queried ASD. Although some features of NET (e.g., psychoeducation, lifeline as a visual medium, structured and repetitive sessions) may be well-suited for autistic young people, further adaptations may be warranted to address additional needs, such as emotion regulation and functioning difficulties.

A further clinical implication of this study is that clinicians offering NET to young people may wish to consider the most appropriate medium of delivery (face-to-face versus videoconferencing) on a case-by-case basis. Even as most services have resumed in-person clinical contacts, clinicians are encouraged to explore young people’s preferences around this, as well as and potential barriers that might prevent them from engaging in online therapy. Considerations may include the young person’s ability to tolerate prolonged screen time, possible impact on rapport building, the therapy potentially taking place in a re-triggering environment, and access to a confidential space. Potential adjustments to the NET protocol to overcome such barriers may be: contracting a greater number of shorter exposure sessions and negotiating breaks during each session, as recommended by Marlow et al. (2022); if possible, adopting a blended delivery approach whereby the initial stage involving psychoeducation and rapport-building with the young person and their caregivers occurs in person, which appears to positively impact the therapeutic relationship when moving to online settings (Worsley et al., 2022); clarifying the client location and whether they are in a confidential space at the beginning of each session; and carefully assessing risk of dissociation, increased emphasis on safety strategies (e.g., therapist to support the young person to assemble an anti-dissociation kit), using a Body scan before and after exposure to identify dissociative responses, increased contrasting between past and present during narrative exposure, and more active enquiry on physiological reactions that are not visible on screen, as highlighted by Robjant et al. (2020).

### Limitations and Implications for Future Research

The study findings need to be seen in light of several limitations. In spite of the authors’ efforts to control for baseline trends when estimating effect sizes, it cannot be completely ruled out that an extended baseline would have revealed a spontaneous remission of symptoms without the intervention, nor that the observed therapeutic changes were not due to non-specific factors (e.g., therapist factors, receiving support while on the waiting list, etc.). Similarly, the results may be explained by pre-existing differences between participants, including their preference for or aversion toward remote interventions. To address these issues, future research may involve a feasibility randomized control trial design comparing face-to-face KIDNET, online KIDNET, and a non-active control. Further research with greater control and larger samples is also needed to answer questions about generality of the present findings to the broader population of DV-exposed young people.

Additional limitations concerned the chosen measures. This study utilized the CRIES-13 to investigate PTSS, however, the CRIES-8, which does not include hyperarousal items, has been shown to perform equally well, if not better, than the 13-item version; this is because intrusion and avoidance symptoms appear to be more predictive of PTSD clinical caseness (Perrin et al., 2005). This might have affected the RC and CSC analyses for the CRIES-13 scores in this study, in that participants’ elevated hyperarousal symptoms at the end of the intervention might have been reflective of other difficulties, such as depression or anxiety, rather than PTSS. Furthermore, the YP-CORE mainly assesses internalizing symptoms and the study did not quantitatively investigate changes in other areas that were highlighted in participants’ clinical records or in the Change Interviews. Therefore, future NET studies with young people may wish to assess areas such as externalizing symptoms, auditory hallucinations/voice hearing, dissociative symptoms, and relational difficulties.

The study short FU period and the number of missing quantitative and qualitative data in this phase made it difficult to draw conclusions regarding the potential effectiveness of video NET in the long term and limited inferences about acceptability. Specifically, the missing quantitative data highlighted a flaw in the implementation of the design, as asking participants to continue completing the outcome measures weekly and for approximately a month after ending video NET appeared to be too burdensome. Future research designs evaluating video NET with young people may involve longer FU periods, but with less frequent measurements/observations, and further explore young people’s experiences of NET.

## Conclusions

Albeit limited, the quantitative and qualitative findings of this study offer preliminary support for the use of video NET with young people with past exposure to DV. In particular, video NET appeared to reduce PTSS and, to a lesser extent, general psychological distress in some DV-affected youth. Changes in the desired direction were also observed for NET main putative mechanisms of change, namely integration of traumatic memories in the majority of participants and WHS in all participants with available data. Some young people in this study also demonstrated BHS. This study also provides initial evidence that video NET may be feasible and acceptable for this client group. Despite the promising findings, this study suggests that additional interventions may be needed to meet this group’s complex clinical needs and that further research with greater control and larger samples should focus on corroborating and expanding on the present results.
